# Synchronous cesarean delivery and revision of infected ventral hernia repair mesh in a complex abdominal wall

**DOI:** 10.1093/jscr/rjae151

**Published:** 2024-09-26

**Authors:** Tiffany Bender, Parker Owen, Kristopher Johnson, Matthew Sorrell, Rachel Rodel

**Affiliations:** University of South Dakota Sanford School of Medicine, Sioux Falls, SD 57105, United States; University of South Dakota Sanford School of Medicine, Sioux Falls, SD 57105, United States; Department of General Surgery, University of South Dakota Sanford School of Medicine, Sioux Falls, SD 57105, United States; Department of General Surgery, Sanford Health, Sioux Falls, SD 57105, United States; Department of Obstetrics and Gynecology, University of South Dakota Sanford School of Medicine, Sanford Health, Sioux Falls, SD 57105, United States

**Keywords:** ventral hernia, cesarean delivery, pregnancy, hernia repair

## Abstract

Ventral hernias are a common abdominal wall defect vulnerable to the gravid abdomen’s physiological changes. This case report describes a 38-year-old gravida 3 para 2002 female with a complex abdominal surgical history and a chronic infection of the abdominal wall at the site of prior hernia repair with mesh. She was managed conservatively with antibiotics until delivery. Abdominal wall debridement and repair was coordinated with her 39-week cesarean, which allowed for a successful delivery of her infant paralleled with surgical management of the infected mesh.

## Introduction

A ventral hernia (VH) is a protrusion of abdominal contents, often bowel, through an anterior abdominal wall [1]. The incidence of VH repairs in the USA has risen, attributed to factors such as obesity and an aging population [2–4]. Increased age, smoking, and diabetes are risk factors for hernia development. Additionally, increases in abdominal pressure from heavy lifting, chronic coughing, severe vomiting, and pregnancy also increase the risk of VH [5].

Severity varies from mild and asymptomatic to painful and life-threatening [1]. Asymptomatic or reducible hernias can be observed longitudinally or electively repaired. Symptomatic cases necessitate surgical repair to avoid complications like incarceration or strangulation [6]. Repair reduces the protrusion of abdominal contents and corrects the structural defect, often with mesh.

A challenging complication is infection, as it often requires complicated repair and results in a long recovery [6, 7]. Few guidelines exist on how to specifically manage infected mesh and options range from conservative therapy to complete removal and revision of the repair [8, 9]. Conservative therapies, such as antibiotics and negative pressure wound therapy, are often effective, particularly in poor surgical candidates [10]. In most pregnant women, it is recommended to postpone surgical repair until delivery [11]. In severe cases, such as incarceration or strangulation, a repair in the second trimester may be justified [12].

This case study examines a patient with mesh infection from a prior VH repair, whose treatment options were limited because of her current gestation and high body mass index (BMI).

## Case presentation

A 38-year-old gravida 3 para 2002 female at 20 weeks gestation was referred to maternal–fetal medicine (MFM) from an outside facility with complaints of abdominal pain and drainage from abdominal scars. Medical history was significant for an elevated BMI of 44.81 kg/m^2^. Her obstetric history consisted of a primary cesarean delivery (CD) with a Pfannenstiel incision for cephalopelvic disproportion and fetal intolerance of labor. During her second pregnancy, she had a repeat CD. Approximately 4 months later, she developed an incarcerated VH and a repair was completed with preperitoneal 15 × 15 cm bioabsorable mesh. The patient developed postoperative drainage acutely and observation was recommended. The drainage persisted for 2–3 years and required dressing changes up to 6 times daily. She was otherwise asymptomatic throughout this time.

**Figure 1:**
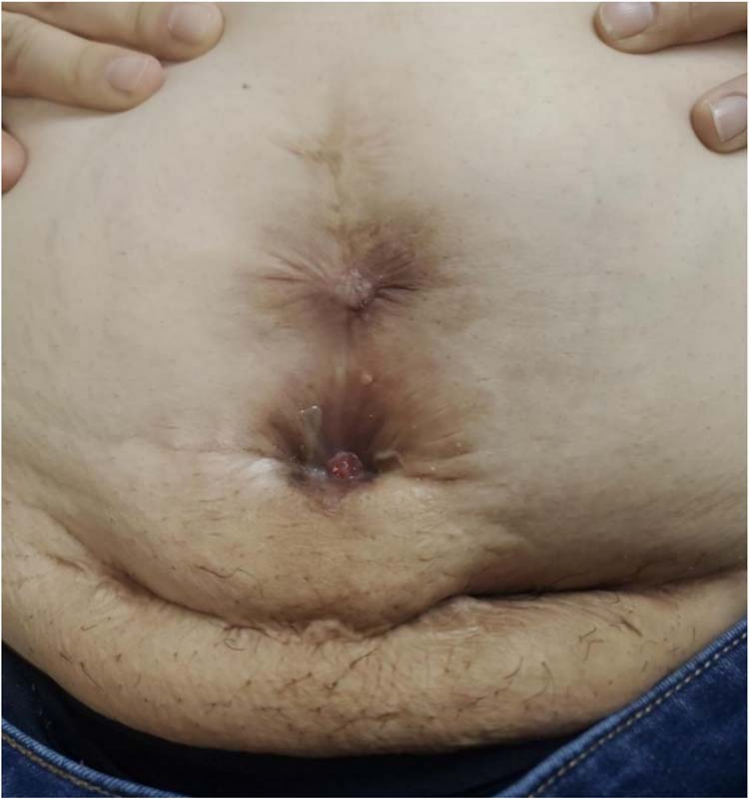
Purulent drainage at the inferior aspect of the vertical scar.

**Figure 2:**
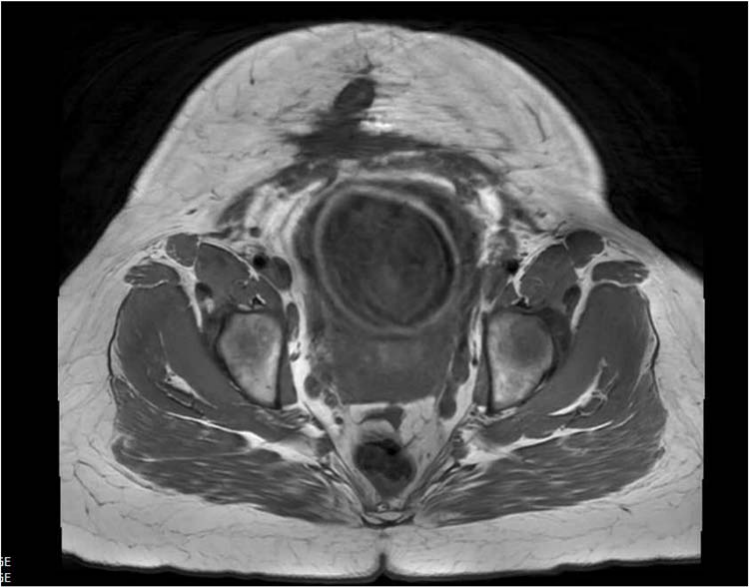
Abdominal MRI scan showing ventral wall defect.

In the first trimester of her third pregnancy, she developed purulent drainage with bleeding and pain at the inferior portion of the vertical scar (Fig. 1). This drainage, in addition to abdominal pain, caused her to seek care. She was admitted for IV antibiotics and discharged home to complete a course of oral antibiotics. She had ongoing interdisciplinary care throughout her pregnancy including her local obstetrician, a consulting MFM specialist, and general surgery (GS). She intermittently received antibiotics for worsening symptoms. An MRI was obtained (Fig. 2) in the third trimester to further characterize the abdominal wall. No abscesses were visualized.

At 39 weeks, a planned interdisciplinary CD was performed. Regional anesthesia was placed, although the level of pain control was not adequate at the time of the initial incision and transition to general anesthesia was made. An elliptical incision was made around the previous midline scar and musculature and fascia were dissected. Multiple sinus tracts with purulence were observed and cultured. The remaining layer of fascia was dissected to expose the uterus. A CD with bilateral salpingectomy was performed without complications. After the CD, the sinus tracts were debrided until normal fascia and scar tissue were achieved. The mesh was incorporated into the abdominal wall; however, the sutures communicated with the sinus tracts, necessitating excision. The surrounding area was debrided until healthy tissue remained. Approximately 60 cm^2^ of muscle, fascia, and mesh were excised.

Despite extensive debridement, primary closure of the fascia was achieved. Subcutaneous tissue flaps were created, facilitating the placement of a 6 × 8 inch synthetic bioabsorbable mesh, secured peripherally with staples and interrupted polyglactin 910 sutures adjacent to the midline incision. A subcutaneous drain was placed within the subcutaneous tissue. The skin was closed with interrupted deep dermal polyglactin 910 sutures and approximated with a skin stapler. A closed negative pressure wound therapy device was placed over the incision.

The patient tolerated the procedure well and returned to the postpartum unit for recovery. Throughout the hospital course, she had a total of 200 mL of thin serosanguineous output from her drain. The cultures of the purulent sinus tracts resulted in a moderate amount of *Staphylococcus aureus* with an intermediate resistance to gentamicin. She was discharged on postoperative Day 2 with a 2-week course of cephalexin and instructions to follow-up in 2 weeks with a local surgeon for staple removal.

 Twenty-eight days after the abdominal wall reconstruction, the patient contacted her local surgeon with worsening lower abdominal pain, umbilical erythema, and fever. Abdominal CT revealed a rim-enhancing fluid collection in the subcutaneous tissue overlying the biological mesh. She was taken to the OR for a subsequent irrigation and debridement (I&D) at the previous incision site with abscess drainage and debridement. The mesh did not require explantation. Approximately two months following the I&D, the patient had resolution of the infection and seroma confirmed with ultrasound.

## Discussion

Managing ventral hernias in pregnant patients lacks structured guidelines, requiring careful risk–benefit assessment. Pregnancy introduces surgical complexities and physiologic changes of the gravid abdomen may worsen hernia defects [13]. This patient’s presentation was complicated by her infected mesh, extensive abdominal scarring, and elevated BMI.

This chronic draining wound necessitated treatment, which was able to be managed conservatively with antibiotics during pregnancy. While antibiotic therapy did not address the anatomic defect, it targeted the soft tissue infection surrounding the mesh until delivery. The decision to collaborate delivery with GS omitted the need for the patient to undergo additional surgery postpartum. Furthermore, this collaboration prevented additional travel and medical burden for a patient who had already experienced barriers to medical care.

## Conclusion

This case highlights the intricate management of infected mesh in a pregnant patient with complex abdominal wall scarring and high BMI. Conservative antibiotic therapy, collaborative delivery planning, and careful consideration of risks were crucial in achieving a successful outcome. The case underscores the importance of individualized approaches in managing complex VH cases during pregnancy.

## Conflict of interest statement

None declared.

## Funding

None declared.
